# Current Status of Endoscopic Biliary Drainage in Patients with Distal Malignant Biliary Obstruction

**DOI:** 10.3390/jcm10194619

**Published:** 2021-10-08

**Authors:** Yuki Tanisaka, Masafumi Mizuide, Akashi Fujita, Tomoya Ogawa, Hiromune Katsuda, Youichi Saito, Kazuya Miyaguchi, Ryuhei Jinushi, Rie Terada, Yuya Nakano, Tomoaki Tashima, Yumi Mashimo, Shomei Ryozawa

**Affiliations:** Department of Gastroenterology, Saitama Medical University International Medical Center, 1397-1, Yamane, Hidaka, Saitama 350-1298, Japan; mizuide1971@yahoo.co.jp (M.M.); a.fujita0628@gmail.com (A.F.); t.ogawa0210@icloud.com (T.O.); goobygoobygoobygoo@gmail.com (H.K.); stm_ys41@yahoo.co.jp (Y.S.); kaz.hr77@gmail.com (K.M.); gk3273@icloud.com (R.J.); katorie0308@gmail.com (R.T.); koijuhygtfrdeswaq@yahoo.co.jp (Y.N.); t.tashima1981@gmail.com (T.T.); ymashimo@saitama-med.ac.jp (Y.M.); ryozawa@saitama-med.ac.jp (S.R.)

**Keywords:** distal malignant biliary obstruction, endoscopic biliary drainage, self-expandable metal stents, covered self-expandable metal stents, laser-cut, endoscopic retrograde cholangiopancreatography, endoscopic ultrasound, interventional endoscopic ultrasound, endoscopic ultrasound-guided biliary drainage

## Abstract

Distal malignant biliary obstruction is caused by various malignant diseases that require biliary drainage. In patients with operable situations, preoperative biliary drainage is required to control jaundice and cholangitis until surgery. In view of tract seeding, endoscopic biliary drainage is the first choice. Since neoadjuvant therapies are being developed, the time to surgery is increasing, especially in pancreatic cancer cases. Therefore, it requires long stent patency. Recently, preoperative biliary drainage using self-expandable metal stents has been reported as a useful modality to secure long stent patency. In patients with unresectable distal malignant biliary obstruction, self-expandable metal stent is the first choice for maintaining long stent patency. Although there are many comparison studies between a covered and an uncovered self-expandable metal stent, their use is still controversial. Recently, endoscopic ultrasound-guided biliary drainage has been performed as an alternative treatment. The clinical success and stent patency are favorable. We should take into consideration that both endoscopic retrograde cholangiopancreatography-guided biliary drainage and endoscopic ultrasound-guided biliary drainage have advantages and disadvantages and chose the drainage method depending on the patient’s situation or the expertise of the endoscopist. Here, we discuss the current status of endoscopic biliary drainage in patients with distal malignant biliary obstruction.

## 1. Introduction

Various malignant diseases, such as cholangiocarcinoma, pancreatic cancer, ampullary cancer, and metastatic cancers, cause distal malignant biliary obstruction. Among undetermined biliary strictures, it is difficult to make a correct diagnosis of distal malignant biliary obstruction; a multimodal approach is required for the correct diagnosis [1]. Distal malignant biliary obstruction causes obstructive jaundice and cholangitis; therefore, appropriate biliary drainage is required. Biliary drainage improves the patient’s quality of life and prevents hepatobiliary dysfunction and liver failure. Although percutaneous transhepatic biliary drainage has been traditionally performed, it may be impractical for urgent cases because of the requirement of serial dilation and track maturation [2]. Moreover, seeding metastasis can occur [3]. Therefore, endoscopic biliary drainage is thought to be the first choice and has been an established procedure.

Endoscopic biliary drainage for patients with distal malignant biliary obstruction is divided into two situations: preoperative biliary drainage and palliative drainage for patients with unresectable cancer. Recently, preoperative biliary drainage using self-expandable metal stents has been reported as a useful modality to secure long stent patency. In patients with unresectable distal malignant biliary obstruction, a self-expandable metal stent is thought to be the first choice for maintaining long stent patency. Moreover, owing to the efficacy of endoscopic ultrasound-guided biliary drainage, it has been reported as an alternative treatment for distal malignant biliary obstruction. As there are various methods and techniques for biliary drainage, an appropriate drainage strategy is warranted. In this review, we discuss the current status of endoscopic biliary drainage in patients with distal malignant biliary obstruction.

## 2. Diagnostic Strategy for Distal Malignant Biliary Obstruction

Before mentioning biliary drainage, as distal malignant biliary obstruction has a poor prognosis, an appropriate diagnostic strategy for distal malignant biliary obstruction is essential [1]. Among distal biliary obstructions, benign strictures, such as chronic pancreatitis, autoimmune pancreatitis, and immunoglobulin G4-associated cholangitis should be considered. It is sometimes difficult to differentiate between benign biliary strictures and distal malignant biliary obstruction.

The initial assessment should be noninvasive, such as those performed using medical examinations and taking the patient’s history. Distal malignant biliary obstruction leads to jaundice (conjunctiva and skin), discolored stools, dark urine, pruritus, nausea, and vomiting. Laboratory tests, such as total bilirubin, alkaline phosphatase, and gamma-glutamyltransferase levels are also performed. As for “tumor markers,” it is widely accepted that testing for the carcinoembryonic antigen and carbohydrate antigen (CA) 19-9, prognostic factors and indicators of tumor resectability, is useful in diagnostics. However, these markers have low positive predictive values, and the levels of CA 19-9 can also increase in other hepatobiliary conditions, including jaundice and cholangitis [4,5,6,7]. Cross-sectional imaging, such as abdominal echo, computed tomography (CT), and magnetic resonance imaging (MRI) are useful. CT or MRI can detect metastatic lesions; therefore, cancer staging can be diagnosed.

After a noninvasive approach, endoscopic approaches, such as endoscopic ultrasound and endoscopic retrograde cholangiopancreatography are performed. Endoscopic ultrasound provides high-resolution findings, and its sensitivity and specificity have been reported to be 78% and 84% [8]. Moreover, histological assessment using endoscopic ultrasound-guided fine-needle aspiration is also useful. The diagnostic accuracy of endoscopic ultrasound-guided fine-needle aspiration has been reported to be approximately 90% [9]. Distal malignant biliary obstruction due to pancreatic cancer is a good indication for endoscopic ultrasound-guided fine-needle aspiration. The advantage of endoscopic ultrasound-related procedures compared to endoscopic retrograde cholangiopancreatography is that it avoids adverse events, such as pancreatitis.

Since 1968, endoscopic retrograde cholangiopancreatography has been considered the gold standard for diagnosis and intervention in biliopancreatic diseases [10]. In endoscopic retrograde cholangiopancreatography, biliary strictures are comprehensively diagnosed using cholangiography, biopsy, or cytology. The diagnostic sensitivity and specificity for cholangiography findings were 74% and 70%, respectively [11]. A recent meta-analysis reported that the sensitivity and specificity of brush cytology was 45% (95% confidence interval (CI) (40–50%)) and 99% (95% CI (98–100%)), respectively, whereas the sensitivity and specificity of forceps biopsy was 48.1% (95% CI (42.8–53.4%)) and 99.2% (95% CI (97.6–99.8%)), respectively [12]. Moreover, endoscopic retrograde cholangiopancreatography enables to perform therapeutic roles such as biliary drainage in the same session. However, it is more invasive than others such as CT, MRI, and endoscopic ultrasound. The recent European Society of Gastrointestinal Endoscopy Guidelines reported that the rates of pancreatitis, cholangitis, and perforation during/post-endoscopic retrograde cholangiopancreatography have been reported to be 3.5–9.7%, 0.5–3.0%, and 0.08–0.6%, respectively. Moreover, the mortality rate of post-endoscopic retrograde cholangiopancreatography pancreatitis has been reported to be 0.1–0.7% [13]. Although endoscopic retrograde cholangiopancreatography is an essential procedure to assess biliary strictures, we must keep in mind that severe and fatal endoscopic retrograde cholangiopancreatography-related adverse events can occur.

There are several additional diagnostic aids for endoscopic retrograde cholangiopancreatography-related procedures. Endoscopic retrograde cholangiopancreatography has a disadvantage that it does not provide an intraluminal view of biliary strictures. Cholangioscopy provides direct visualization of the biliary tract. Moreover, forceps biopsy under the direct view of cholangioscopy is possible [14]. It was reported that the pooled sensitivity and specificity for diagnosing malignancy by cholangioscopy-guided biopsy were 60.1% (95% CI (54.9–65.2%)) and 98.0% (95% CI (96.0–99.0%)), respectively [15].

**Figure 1:**
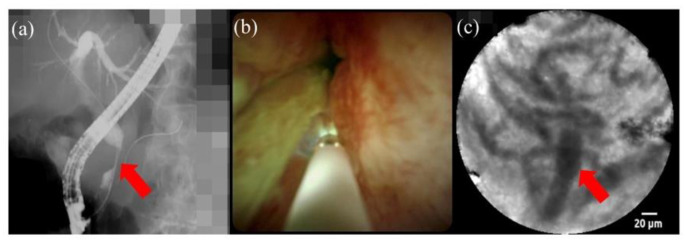
Diagnosis using confocal laser endomicroscopy under direct view of cholangioscopy. (**a**) Cholangiography shows a distal biliary stricture (red arrow). (**b**) Cholangioscopy shows a reddish papillogranular surface. (**c**) Probe-based confocal laser endomicroscopy shows a thickened reticular structure indicating inflammation.

Confocal laser endomicroscopy uses a low-power laser to create real-time high-resolution and magnified images of the mucosal layer of the gastrointestinal tract. Probe-based confocal laser endomicroscopy has been mentioned in the recent American Society for Gastrointestinal Endoscopy guidelines for the management of biliary neoplasia as a useful method [16]. Combining the endoscopic retrograde cholangiopancreatography impression with probe-based confocal laser endomicroscopy findings, it was reported that sensitivity and specificity were 89% and 71%, respectively, in a prospective study of 112 patients [17]. Although probe-based confocal laser endomicroscopy can be performed under fluoroscopy guidance or direct view of cholangioscopy, the probe-based confocal laser endomicroscopy findings under direct view of cholangioscopy can be accurately matched with those of biopsy [18] (Figure 1).

## 3. Preoperative Biliary Drainage for Patients with Distal Malignant Biliary Obstruction

Routine endoscopic preoperative biliary drainage for patients with distal malignant biliary obstruction is supposed to increase the rate of complications; thus, it is not generally recommended [19,20]. In some studies on preoperative biliary drainage, an increased mortality rate or a high frequency of surgical site infection have been observed [21,22]. A recent randomized controlled trial showed that preoperative biliary drainage is associated with an increased incidence of perioperative adverse events [23]. In this study, 202 patients with resectable pancreatic head cancer underwent either an early surgery within 1 week without preoperative biliary drainage or endoscopic retrograde cholangiopancreatography with preoperative biliary drainage, 7-Fr plastic stent placement, and a delayed surgery 4–6 weeks later. Although this study suggested the possibility of demerits regarding preoperative biliary drainage, we should consider that the initial procedure failure rate was 25%, which is higher than that reported in other studies. Moreover, patients with severe jaundice (>14.6 mg/dL) were excluded from the study. Hence, there are still various clinical situations where preoperative biliary drainage may be necessary, such as cholangitis, obstructive jaundice, and long waiting time for surgery. Recently, surgery has become increasingly delayed when neoadjuvant chemotherapy is employed; therefore, the indication for preoperative biliary drainage is also increasing. Percutaneous transhepatic biliary drainage is an effective drainage technique under fluoroscopic guidance for biliary access. A needle is passed through the skin into a dilated biliary duct, after which patients may undergo external drainage [24]. Despite high success rates, it may be impractical for urgent cases because of the requirement of serial dilation and track maturation [2]. The most common complication is tumor seeding along the catheter tract [3]. Therefore, endoscopic biliary drainage is considered as the first choice.

**Figure 2:**
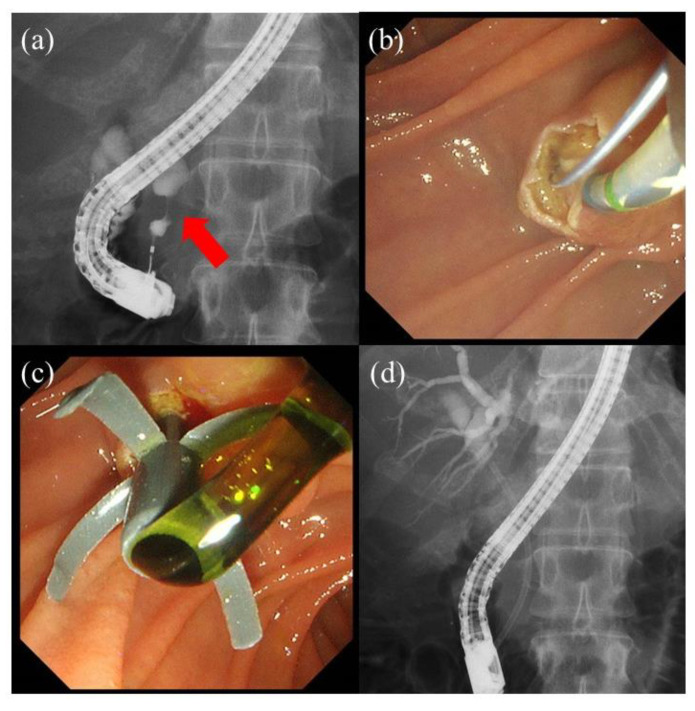
A case of preoperative biliary drainage for a patient with distal malignant biliary obstruction. (**a**) Cholangiography shows a distal biliary stricture (red arrow). (**b**) Endoscopic sphincterotomy is performed. (**c**,**d**) An 8.5 Fr 7 cm plastic stent is placed.

Endoscopic biliary drainage is performed during the preoperative endoscopic retrograde cholangiopancreatography-related procedures. After cholangiography, an intraductal ultrasound is performed to detect the main lesion and extent of the lesion. Endoscopic sphincterotomy is performed as necessary. After forceps biopsy or cytology, endoscopic biliary drainage is finally performed. Plastic stent placement has been the standard treatment for preoperative biliary drainage in patients with distal malignant biliary obstruction, especially in those not undergoing neoadjuvant chemotherapy [25]. The diameter of the plastic stent is ordinally from 7 to 10 Fr (Figure 2). Although some studies reported that a 10 Fr plastic stent had longer patency than a 7 Fr plastic stent (3–5 months versus 8 weeks) [26,27], we sometimes experience recurrent biliary obstruction owing to stent occlusion and stent migration in a few weeks even with a 10 Fr plastic stent placement.

Self-expandable metal stent placement has been reported to have a higher patency rate, lower incidence of complications, and greater cost-effectiveness than plastic stent placement in patients with unresectable distal malignant biliary obstruction [28,29,30,31]. Recently, preoperative biliary drainage using self-expandable metal stents has been reported as a useful modality to secure long stent patency, especially in patients undergoing neoadjuvant chemotherapy for pancreatic cancer, because patency rates depend on the luminal diameter of the stent [19,32]. Regarding self-expandable metal stent placement, surgeons are concerned that self-expandable metal stents may cause local inflammation and adhesion around the bile duct, which may interfere with surgical resection. Recent data showed that self-expandable metal stent placement, at least 2 cm below the hilum, was not associated with technical difficulties and did not affect the outcomes of the surgery [33].

Table 1 shows the outcomes of preoperative biliary drainage between plastic stent and self-expandable metal stent placement in patients with distal malignant biliary obstruction [34,35,36,37,38,39,40]. Among the seven studies in Table 1, the rate of recurrent biliary obstruction in the plastic stent and self-expandable metal stent groups was 3.5–3.5% and 0–30.3%. Four studies showed that the rate of recurrent biliary obstruction was significantly lower in the self-expandable metal stent group than in the plastic stent group [35,38,39,40]. Moreover, four studies reported that stent patency was significantly longer in the self-expandable metal stent group than plastic stent groups [34,36,39,40]. As the distal malignant biliary obstruction had only distal bile duct stricture, biliary drainage success rate was almost all 100% in both plastic stent and metallic stent groups. In view of cost, one study [34] showed that metallic stent took more cost, statistically significant compared to plastic stent. Other studies had no significant difference between two groups. Although the plastic stent group showed lower cost per procedure, plural procedures were more likely needed due to stent occlusion, so some studies showed higher total cost than the metallic stent group. If the period of waiting time to surgery is long, it could cost more for use of plastic stent. There was no significant difference between plastic stent and metallic stent regarding adverse events. Main adverse events between two groups were pancreatitis. Although these reports showed the usefulness and safety of self-expandable metal stent placement for patients with distal malignant biliary obstruction preoperatively, further randomized control studies with a large number of patients are warranted.

## 4. Palliative Biliary Drainage for Patients with Unresectable Distal Malignant Biliary Obstruction

**Figure 3:**
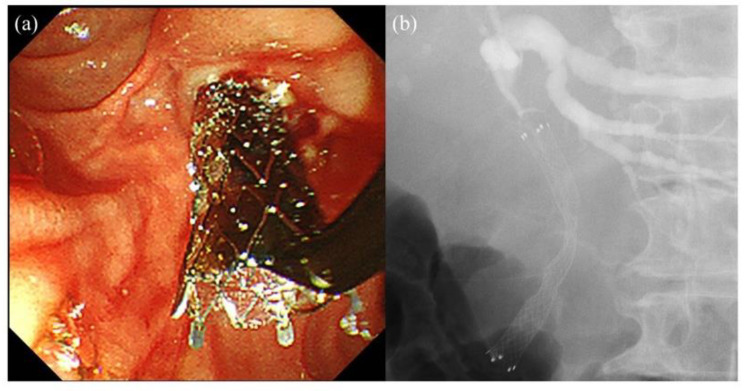
A case of palliative biliary drainage for a patient with unresectable distal malignant biliary obstruction. (**a**,**b**) A laser-cut covered self-expandable metal stent (10 mm diameter, 8 cm length; X-Suit NIR covered biliary metal stent; Olympus Medical Systems, Tokyo, Japan) is placed across the papilla.

Despite the progression of the diagnosis process and surgery, distal malignant biliary obstruction has no curative perspective at the time of diagnosis in many cases. Thus, palliative treatment to achieve bile duct clearance plays a major role in providing a long life expectancy and improved quality of life. The usefulness of self-expandable metal stent placement for patients with unresectable distal malignant biliary obstruction has been reported in many studies; therefore, it is an established procedure [28,29,30,31]. Thus, many facilities perform self-expandable metal stent placement in such cases, except for patients with a short prognosis. The self-expandable metal stent is divided into a covered self-expandable metal stent and an uncovered self-expandable metal stent. The merit of the covered self-expandable metal stent is that it prevents tumor ingrowth and is easy to remove, while the uncovered self-expandable metal stent is thought to prevent stent migration and acute pancreatitis due to self-expandable metal stent compression [41,42,43,44]. Although there are some reports that a 12 mm self-expandable metal stent is useful for patients with unresectable distal malignant biliary obstruction [45,46], an 8–10 mm diameter of the self-expandable metal stent is generally used (Figure 3). As the self-expandable metal stent has a larger diameter than the plastic stent, endoscopic sphincterotomy is performed as necessary to prevent acute pancreatitis due to self-expandable metal stent compression. A randomized control study of 200 patients with distal malignant biliary obstruction caused by unresectable pancreatic cancer in 25 facilities reported that endoscopic sphincterotomy did not affect the outcomes of self-expandable metal stent placement procedures, including the occurrence of procedure-related pancreatitis [47]. In patients with pancreatic cancer, exocrine function has already ceased because of a main pancreatic duct obstruction; therefore, it was thought that endoscopic sphincterotomy was not associated with procedure-related pancreatitis in this study. However, in any other distal malignant biliary obstruction, such as cholangiocarcinoma, endoscopic sphincterotomy should be performed to prevent pancreatitis.

**Figure 4:**
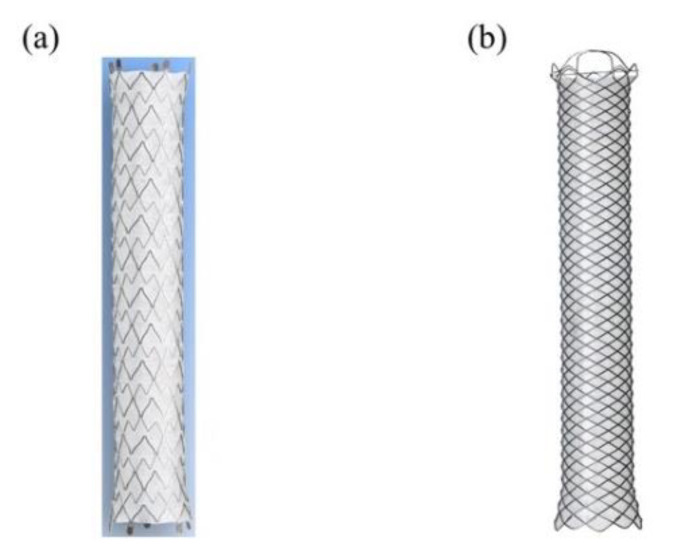
Covered self-expandable metal stents. (**a**) Laser-cut-covered self-expandable metal stent; X-Suit NIR covered biliary metal stent (Olympus Medical Systems, Tokyo, Japan). (**b**) Braided-covered self-expandable metal stent; WallFlex biliary RX fully covered stent (Boston Scientific, Natick, MA, USA).

Moreover, the self-expandable metal stent is mainly classified into two types based on its structure: braided-self-expandable metal stent and laser-cut-self-expandable metal stent (Figure 4). The braided self-expandable metal stent has a crisscross mesh structure, whereas the laser-cut self-expandable metal stent does not; however, it is connected by thin and thick struts. Hence, the braided self-expandable metal stent has an approximately 40% shortening rate, while the laser-cut-self-expandable metal stent has minimal stent shortening. We should place the braided-self-expandable metal stent or laser-cut-self-expandable metal stent while considering stent shortening. Although the advantage of the covered self-expandable metal stent is easy removal, it is thought that performing endoscopic removal of the laser-cut-self-expandable metal stent in cases of recurrent biliary obstruction is difficult due to its stent structure compared to that of the braided-covered self-expandable metal stent. However, recent studies have shown the possibility of endoscopic removal of the laser-cut self-expandable metal stent [48,49,50,51]. Moreover, the newly developed laser-cut-self-expandable metal stent, which has an anti-reflux valve to prevent duodenobiliary reflux, has been reported to be useful for patients with unresectable distal malignant biliary obstruction [52,53]. This may contribute to the reduction in recurrent biliary obstruction due to duodenobiliary reflux. Although there is still a lack of evidence regarding the laser-cut-self-expandable metal stent, it is easy to place it in the intended location of the bile duct; thus, further studies to prove its efficacy and safety are warranted.

Table 2 shows the outcomes of biliary drainage for patients with unresectable distal malignant biliary obstruction between the covered self-expandable metal stent and uncovered self-expandable metal stent [41,42,54,55,56]. Although only one study [41] showed that the rate of recurrent biliary obstruction was significantly higher in the uncovered self-expandable metal stent group than in the covered self-expandable metal stent group, other studies showed no significant difference. Regarding the time to recurrent biliary obstruction, two studies [41,42] showed that the covered self-expandable metal stent group, while one study showed [56] that the uncovered self-expandable metal stent group had long stent patency. There was no significant difference between covered metallic stent and uncovered metallic stent regarding adverse events. Main adverse events between two groups were pancreatitis and cholecystitis. In spite of using uncovered metallic stent, pancreatitis and cholecystitis occurred similar to use of covered metallic stent. A recent systematic review and meta-analysis that identified only randomized control studies (1272 patients in 11 studies) showed that recurrent biliary obstruction and patient mortality did not differ significantly between the covered self-expandable metal stent and uncovered self-expandable metal stent, but stent migration and sludge formation occurred frequently with the covered self-expandable metal stent. Moreover, the covered self-expandable metal stent had a lower rate of tumor ingrowth but a higher rate of tumor overgrowth compared to the uncovered self-expandable metal stent [57]. Although there are many comparison studies between the covered self-expandable metal stent and uncovered self-expandable metal stent, their use is still controversial. Further improvements in both the covered self-expandable metal stent and uncovered self-expandable metal stent are required.

## 5. Endoscopic Ultrasound-Guided Biliary Drainage

Endoscopic retrograde cholangiopancreatography-related procedures have been reported to be successful in approximately 95% of cases [10,58]. However, it is sometimes difficult to complete the procedure in many situations, such as difficult biliary cannulation and surgically altered anatomy [59,60]. Moreover, distal malignant biliary obstruction could cause duodenal obstruction due to invasion; therefore, it is impossible to reach the papilla in such a situation. Recently, endoscopic ultrasound-guided biliary drainage has been in the spotlight as an alternative therapy for patients with difficult endoscopic retrograde cholangiopancreatography.

**Figure 5:**
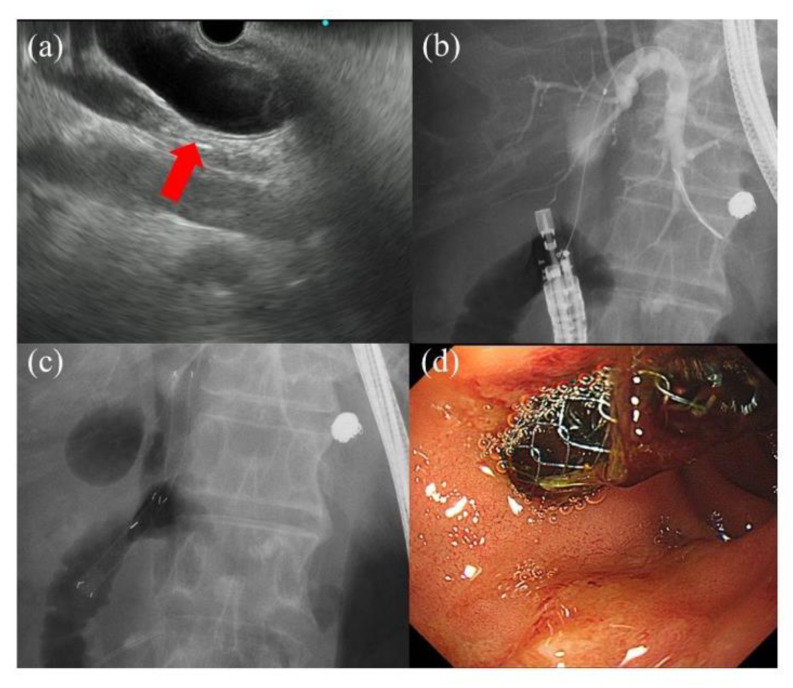
A case of endoscopic ultrasound-guided biliary drainage for a patient with distal malignant biliary obstruction. (**a**) Endoscopic ultrasound shows the dilation of the common bile duct (red arrow) (CBD). (**b**) After puncturing the CBD, successful cholangiography is performed. (**c**,**d**) A braided-covered self-expandable metal stent (10 mm diameter, 8 cm length; WallFlex biliary RX fully covered stent; Boston Scientific, Natick, MA, USA) is placed.

There are several drainage methods for interventional endoscopic ultrasound [61]. (1) endoscopic ultrasound-guided choledochoduodenostomy, (2) endoscopic ultrasound-guided hepaticogastrostomy, (3) endoscopic ultrasound-guided anterograde stenting, (4) endoscopic ultrasound-guided rendezvous procedure. The duodenum and stomach are punctured in endoscopic ultrasound-guided choledochoduodenostomy and endoscopic ultrasound-guided hepaticogastrostomy, respectively. After cholangiography and guidewire insertion, the fistula is dilated using a dilation device followed by placement of a biliary stent (Figure 5) [62]. In endoscopic ultrasound-guided anterograde stenting, after puncture of the bile duct, a guidewire is directed to the papilla, and the biliary stent is placed via an antegrade route [63]. An endoscopic ultrasound-guided rendezvous procedure is performed in difficult cannulation cases. After puncture of the bile duct, the guidewire is directed beyond the papilla. As a result, the guidewire is positioned in the duodenum. Afterward, the scope is exchanged with the duodenoscope. The guidewire is grasped using a forceps device and pulled into the working channel. Finally, biliary cannulation through the papilla is successful [64].

Table 3 shows the outcomes of endoscopic ultrasound-guided biliary drainage in patients with distal malignant biliary obstruction [65,66,67,68]. While technical and clinical success rates were high, the rate of adverse events was also high. Focal peritonitis due to bile leak was characteristic of endoscopic ultrasound-guided biliary drainage. A recent systematic review and meta-analysis reported that the pooled technical success rates and the clinical success rates were 91.5% and 87%, respectively. Adverse events occurred in 17.9% of patients. The main adverse events were bile leakage (4.1%), stent migration (3.9%), and infection (3.8%) [69]. Stent migration could cause emergency surgery because bile leak could continue from the fistula created by endoscopic ultrasound-guided biliary drainage. Therefore, endoscopic ultrasound-guided biliary drainage should be performed carefully, and endoscopists should consider the development of severe adverse events.

A recent systematic review and meta-analysis compared endoscopic retrograde cholangiopancreatography-biliary drainage and endoscopic ultrasound-guided biliary drainage [70]. The technical and clinical success rates of endoscopic retrograde cholangiopancreatography-biliary drainage and endoscopic ultrasound-guided biliary drainage were 96.7% (404/418) versus 96.3% (208/216) and 93.2 (342/367) versus 96.3% (180/187), respectively. There were no significant differences between the two groups. The rate of adverse events between endoscopic retrograde cholangiopancreatography-biliary drainage and endoscopic ultrasound-guided biliary drainage was also not significantly different between the two groups (16.3% (62/380) versus 13.8% (27/196)). The reintervention rates between endoscopic retrograde cholangiopancreatography-biliary drainage and endoscopic ultrasound-guided biliary drainage were 17.5% (31/177) and 5.7% (7/122), respectively. The reintervention rate was significantly low in the endoscopic ultrasound-guided biliary drainage group. Although recent advances in techniques and devices regarding endoscopic ultrasound-guided biliary drainage seem to be effective and safe, those results were from experts of endoscopic ultrasound-guided biliary drainage; therefore, an appropriate procedure, whether endoscopic retrograde cholangiopancreatography-biliary drainage or endoscopic ultrasound-guided biliary drainage, for patients with distal malignant biliary obstruction should be chosen based on the patient’s condition or the expertise of the endoscopist.

## 6. Conclusions

We discussed the current status of endoscopic biliary drainage in patients with distal malignant biliary obstruction. As we mentioned, among distal biliary obstructions, benign diseases could be included; therefore, the correct diagnosis before biliary drainage is very important. If it is difficult to make a correct diagnosis, advanced modalities, such as cholangioscopy and probe-based confocal laser endomicroscopy should be used. In cases of preoperative biliary drainage, the choice of a plastic stent or self-expandable metal stent should depend on the period of waiting time to surgery. If surgery could be performed within a few weeks, plastic stent placement should be preferred in view of the medical cost. If awaiting surgery would be over 1 month, self-expandable metal stent placement should be considered. A discussion with the surgeon is important in selecting the biliary stent. In cases of palliative biliary drainage for patients with unresectable distal malignant biliary obstruction, the choice of endoscopic retrograde cholangiopancreatography-biliary drainage or endoscopic ultrasound-guided biliary drainage should depend on the patient’s condition or the expertise of the endoscopist. Endoscopic ultrasound-guided biliary drainage could be preferred in cases of duodenal strictures. In cases of endoscopic retrograde cholangiopancreatography-biliary drainage, self-expandable metal stent placement is a good indication for patients whose prognosis is expected to be over 2 months. As it is still controversial whether the covered self-expandable metal stent or uncovered self-expandable metal stent is better, further improvement of both the covered self-expandable metal stent and uncovered self-expandable metal stent is required.

## Figures and Tables

**Table 1:** Outcomes of preoperative biliary drainage between plastic stent and self-expandable metal stent placement in patients with distal malignant biliary obstruction.

Authors	Year	Number of PS and SEMS Placement	Biliary Drainage Success, % (*n*)	The Rate of Recurrent Biliary Obstruction, % (*n*)	Mean Total Cost (US dollar)	Procedure Related Adverse Events Rate, % (*n*)	Stent Patency
Gardner et al. [34]	2016	21 (PS) 33 (SEMS)	100 (21/21) (PS) 100 (33/33) (SEMS)	52.4 (11/21) (PS) 30.3 (10/33) (SEMS), *p* = 0.15	18,701(PS) 24,874 (SEMS), *p* < 0.01	0 (0/21) (PS) 18.2 (6/33) (pancreatitis 6) (SEMS), *p* = 0.12	Longer in SEMS than PS, *p* < 0.01
Tsuboi et al. [35]	2016	11 (PS) 9 (SEMS)	100 (11/11) (PS) 100 (9/9) (SEMS)	72.7 (8/11) (PS) 0 (0/9), (SEMS), *p* = 0.001	13,650 (PS) 10,580 (SEMS), *p* = 0.19	0 (0/11) (PS), 0 (0/9)	Longer in SEMS than PS, *p* = 0.012
Nakamura et al. [36]	2019	26 (PS) 17 (SEMS)	100 (26/26) (PS) 100 (17/17) (SEMS)	34.6 (9/26) (PS) 17.6 (3/17) (SEMS), *p* = 0.31	7175 (PS) 7680 (SEMS)	N/A	Longer in SEMS than PS, *p* = 0.042
Cho et al. [37]	2020	26 (PS) 27 (SEMS)	96.2 (25/26) (PS) 100 (27/27) (SEMS)	3.8 (1/26) (PS) 3.8 (1/27) (SEMS), *p* > 0.99	N/A	23.1 (6/26) (pancreatitis 5, cholangitis 1) (PS) 22.2 (6/27) (pancreatitis 5) (SEMS), *p* > 0.99	No significant difference, *p* = 0.551
Kuwatani et al. [38]	2020	12 (PS) 17 (SEMS)	100 (12/12) (PS) 100 (17/17) (SEMS)	83.3 (10/12) (PS) 5.9 (1/17) (SEMS), *p* < 0.001	5700 (PS) 4973 (SEMS)	0 (0/12) (PS) 0 (0/17) (SEMS)	Longer in SEMS than PS, *p* < 0.01
Tamura et al. [39]	2021	11 (PS) 11 (SEMS)	100 (11/11) (PS) 100 (11/11) (SEMS)	72.8 (8/11) (PS) 18.2 (2/11) (SEMS), *p* = 0.015	8722 (PS) 7038 (SEMS), *p* = 0.79	63.6 (7/11) (cholangitis 7, cholecystitis 1, liver abscess 1) (PS) 18.2 (2/11) (cholangitis 1, cholecystitis 2) (SEMS), *p* = 0.08	Longer in SEMS than PS, *p* = 0.02
Hasegawa et al. [40]	2021	40 (PS) 27 (SEMS)	100 (40/40) (PS) 100 (27/27) (SEMS)	97.5 (39/40) (PS) 14.8 (4/27) (SEMS), *p* < 0.001	N/A	15.0 (6/40) (pancreatitis 4, cholecystitis 2) (PS), 7.4 (2/27) (pancreatitis 1, cholecystitis 1) (SEMS), *p* = 0.46	Longer in SEMS than PS, *p* < 0.001

PS, plastic stent; SEMS, self-expandable metal stent; *n*, number.

**Table 2:** Outcomes of biliary drainage for patients with unresectable distal malignant biliary obstruction between the covered self-expandable metal stent and uncovered self-expandable metal stent.

Authors	Year	Number of SEMS Placement	Procedure Related Adverse Events Rate, % (*n*)	The Rate of RBO, % (n)	Time to RBO, Days
Isayama et al. [41]	2004	57 (CSEMS) 55 (USEMS)	12.3 (7/57) (pancreatitis 5, cholecystitis 2) (CSEMS) 5.5 (3/55) (pancreatitis 1, hemorrhage 2) (USEMS), *p* = 0.32	14.0 (8/57) (CSEMS) 38.2 (21/55) (USEMS), *p* < 0.001	304 (CSEMS) 161 (USEMS) (mean time), *p* = 0.007
Telford et al. [54]	2010	68 (CSEMS) 61 (USEMS)	4.4 (3/68) (cholecystitis 3) (CSEMS) 6.6 (4/61) (pancreatitis 1, cholecystitis 3) (USEMS), *p* = 0.71	29.4 (20/68) (CSEMS) 18.0 (11/61) (USEMS), *p* = 0.15	357 (CSEMS) 711 (USEMS) (median time), *p* = 0.53
Kullman et al. [55]	2010	188 (CSEMS) 191 (USEMS)	7.5 (14/188) (pancreatitis 3, cholangitis 8, cholecystitis 2, perforation 1) (CSEMS) 10.5 (20/191) (pancreatitis 4, cholangitis 12, cholecystitis 2, hemorrhage 1, perforation 1) (USEMS), *p* = 0.37	25.0 (47/188) (CSEMS) 23.6 (45/191) (USEMS), *p* = 0.81	154 (CSEMS) 199 (USEMS) (first quartile time), *p* = 0.53
Kitano et al. [42]	2013	60 (CSEMS) 60 (USEMS)	3.3 (2/60) (pancreatitis 1, cholecystitis 1) (CSEMS) 3.3 (2/60) (cholecystitis 2) (USEMS), *p* > 0.99	23.3 (14/60) (CSEMS) 36.3 (22/60) (USEMS), *p* = 0.08	583 (CSEMS) 314 (USEMS) (median time), *p* = 0.019
Lee et al. [56]	2014	20 (CSEMS) 20 (USEMS)	5.0 (1/20) (cholecystitis 1) (CSEMS) 0 (0/20) (USEMS), *p* > 0.99	50.0 (10/20) (CSEMS) 20.0 (4/20) (USEMS), *p* = 0.10	207.5 (CSEMS) 413.3 (USEMS) (mean time), *p* = 0.041

CSEMS, covered self-expandable metal stent; USEMS, uncovered self-expandable metal stent; RBO, recurrent biliary obstruction; *n*, number.

**Table 3:** Outcomes of endoscopic ultrasound-guided biliary drainage for patients with distal malignant biliary obstruction.

Authors	Year	Number of Patients	Technical Success Rate% (n)	Clinical Success Rate% (*n*)	Procedure Related Adverse Events Rate% (*n*)
Hara et al. (65)	2011	18	94 (17/18)	100 (17/17)	17 (3/18) (focal peritonitis 2, bleeding 1)
Song et al. (66)	2012	15	86.7 (13/15)	100 (13/13)	23.1(3/13) (focal peritonitis 2, cholangitis 1)
Kunda et al. (67)	2016	57	98.2 (56/57)	94.7 (54/57)	7.1 (4/56) (perforation 2, bleeding 1, cholangitis 1)
Lu et al. (68)	2017	24	95.8 (23/24)	100 (23/23)	13 (3/23) (bleeding 2, cholangitis 1)

*n*, number.

## Data Availability

Data sharing not applicable.
